# Genomic Analysis Reveals Multi-Drug Resistance Clusters in Group B *Streptococcus* CC17 Hypervirulent Isolates Causing Neonatal Invasive Disease in Southern Mainland China

**DOI:** 10.3389/fmicb.2016.01265

**Published:** 2016-08-15

**Authors:** Edmondo Campisi, Roberto Rosini, Wenjing Ji, Silvia Guidotti, Maricarmen Rojas-López, Guozhu Geng, Qiulian Deng, Huamin Zhong, Weidong Wang, Haiying Liu, Cassandra Nan, Immaculada Margarit, C. D. Rinaudo

**Affiliations:** ^1^GlaxoSmithKline Vaccines S.r.l., SienaItaly; ^2^Laboratory of Bacterial Pathogenesis and Immunology, Rockefeller University, New York, NYUSA; ^3^Department of Pharmacy Administration and Clinical Pharmacy, School of Pharmacy, Health Science Center, Xi’an Jiaotong UniversityXi’an, China; ^4^Shanghai Hengrui Pharmaceutical Co. Ltd, ShanghaiChina; ^5^Clinical Laboratory, Guangzhou Women and Children’s Medical Center, Guangzhou Medical University, GuangzhouChina; ^6^Changsha Hospital for Maternal and Child Health, ChangshaChina; ^7^GlaxoSmithKline Pharma, StevenageUK

**Keywords:** *Streptococcus agalactiae*, whole-genome sequencing (WGS), pilus island (PI), integrative conjugative element (ICE), antimicrobial resistance, resistome, clonal complex (CC), sequence type (ST)

## Abstract

Neonatal invasive disease caused by group B *Streptococcus* (GBS) represents a significant public health care concern globally. However, data related to disease burden, serotype distribution, and molecular epidemiology in China and other Asian countries are very few and specifically relative to confined regions. The aim of this study was to investigate the genetic characteristics of GBS isolates recovered from neonates with invasive disease during 2013–2014 at Guangzhou and Changsha hospitals in southern mainland China. We assessed the capsular polysaccharide type, pilus islands (PIs) distribution and *hvgA* gene presence in a panel of 26 neonatal clinical isolates, of which 8 were recovered from Early Onset Disease and 18 from Late Onset Disease (LOD). Among 26 isolates examined, five serotypes were identified. Type III was the most represented (15 cases), particularly among LOD strains (*n* = 11), followed by types Ib (*n* = 5), V (*n* = 3), Ia (*n* = 2) and II (*n* = 1). We performed whole-genome sequencing analysis and antimicrobial susceptibility testing on the 14 serotype III isolates belonging to the hypervirulent Clonal Complex 17 (serotype III-CC17). The presence of PI-2b alone was associated with 13 out of 14 serotype III-CC17 strains. Genome analysis led us to identify two multi-drug resistance gene clusters harbored in two new versions of integrative and conjugative elements (ICEs), carrying five or eight antibiotic resistance genes, respectively. These ICEs replaced the 16 kb-locus that normally contains the PI-1 operon. All isolates harboring the identified ICEs showed multiple resistances to aminoglycoside, macrolide, and tetracycline antibiotic classes. In conclusion, we report the first whole-genome sequence analysis of 14 GBS serotype III-CC17 strains isolated in China, representing the most prevalent lineage causing neonatal invasive disease. The acquisition of newly identified ICEs conferring multiple antibiotic resistance could in part explain the spread of this specific clone among Chinese neonatal isolates and underlines the need for a constant epidemiological surveillance.

## Introduction

In the 1970s *Streptococcus agalactiae* (group B *Streptococcus*, GBS) emerged as predominant pathogen causing sepsis and meningitis in neonates and infants younger than 3 months in the United States and other Western countries (Baker, 2013). GBS can also cause invasive disease in adult patients with underlying medical conditions (Skoff et al., 2009). Asymptomatic GBS colonization of the genital and gastrointestinal tracts of up to 30% of healthy women is the primary risk factor for Early Onset Disease (EOD) occurring in newborns within the 6 days of life (Heath and Schuchat, 2007). The neonate is usually infected by exposure to GBS during delivery, and bacteria can rapidly spread into the bloodstream with rapid appearance of clinical signs like pneumonia, sepsis, or meningitis (Simonsen et al., 2014). Infections occurring in infants between 7 and 89 days of age are designated Late Onset Disease (LOD) and are characterized by meningitis in up to 50% of cases (Joubrel et al., 2015). The introduction of national guidelines recommending universal screening of pregnant women for GBS carriage and use of intrapartum antibiotic prophylaxis during delivery has significantly reduced the incidence of EOD (Puopolo et al., 2005; Melin, 2011). However, these guidelines have no effect on LOD incidence and their implementation is not feasible in most low and middle-income countries (Dagnew et al., 2012; Johri et al., 2013). Alternative strategies, such as maternal immunization are under evaluation (Melin and Efstratiou, 2013; Nuccitelli et al., 2015; Donders et al., 2016).

The capsular polysaccharide (CPS) that GBS expresses into 10 antigenically unique types (Ia, Ib, and II–IX) represents a major virulence factor and vaccine target for its ability to induce protective immunity (Edwards, 2008). Additionally, several surface proteins such as the components of filamentous pilus polymers extending outside the bacterial surface have been identified as important virulence factors and promising vaccine candidates (Maione et al., 2005; Johri et al., 2006). In GBS, three pilus variants (type 1, 2a, and 2b) were described, and all strains carry at least one variant (Margarit et al., 2009).

GBS disease in all age groups is mainly caused by five capsular types (Ia, Ib, II, III, and V) and the serotype III accounts for about 50% of neonatal disease globally (Edmond et al., 2012; Le Doare and Heath, 2013). In particular, type III isolates belonging to the sequence type (ST) 17 lineage account for about 60% of LOD meningitis cases, compared to less than 10% of colonizing isolates (Manning et al., 2009; Joubrel et al., 2015). Due to its epidemiological relevance, the type III/ST-17 has been defined as hypervirulent and its high invasiveness is presumably associated to additional virulence factors besides the CPS (Jones et al., 2006). Genome-based studies described this clone as a homogeneous cluster of strains of recent origin displaying a conserved specific pattern of genes encoding secreted and surface proteins, including the pilus 1 and pilus 2b operons and the Gbs2018C/*hvgA* gene (Brochet et al., 2006; Springman et al., 2014). The Gbs2018C/*hvgA* gene codes for a ST-17 specific surface-anchored protein called hypervirulent GBS adhesin (HvgA) and can be used to specifically identify GBS ST-17 strains (Lamy et al., 2006; Tazi et al., 2010). Functional characterization of this protein has shown that its expression *in vivo* is associated with GBS hypervirulence, as HvgA was required for intestinal colonization and translocation across the intestinal and the blood–brain barrier, and the onset of meningitis (Tazi et al., 2010).

While the burden of GBS disease in infants has been widely investigated in Western countries, only few studies have been carried out so far in Eastern countries, including China (Dagnew et al., 2012; Johri et al., 2013; Wang et al., 2015a). A GBS invasive disease rate of 0.28 per 1000 live births was estimated in a prospective study conducted in Southern mainland China, and four serotypes (Ia, Ib, III, and V) were associated with the identified cases (Liu et al., 2015). A recent analysis of GBS neonatal isolates recovered from 2008 to 2013 in Beijing and Shenzhen reported similar serotype distribution, with a clear prevalence of type III/CC17 strains (Wang et al., 2015a). Particularly high rates of antibiotic resistance have been detected among isolates circulating in China both in infected neonates (Wang et al., 2015a) and in carrier women (Lu et al., 2014; Wang et al., 2015b).

Here, we investigated the genetic characteristics of 26 GBS isolates recovered at Guangzhou and Changsha hospitals from neonates with invasive disease during 2013–2014, and selected 14 serotype III strains belonging to the CC17 for further whole genome sequencing (WGS) analysis, with particular attention to their antibiotic resistome.

## Materials and Methods

### Bacterial Strains, Media, and Growth Conditions

The strain collection analyzed in this study included 26 GBS clinical isolates recovered in China during 2013–2014 from neonates with invasive disease, mostly in Guangzhou hospital and one isolate in Changsha. Eight strains were prospectively isolated from cases enrolled during a former observational study (Liu et al., 2015) while 16 isolates were retrospectively collected in the same region. GBS COH1, a clinical isolate obtained from an infected newborn with sepsis at the Children’s Orthopedic Hospital (COH; Wilson and Weaver, 1985) was used as reference serotype III-ST17 strain in all performed assays. All GBS isolates used in this study and their relative information are listed in **Table 1**. A GBS case was identified when an infant younger than 3 months was GBS-culture positive from a sterile site (blood or cerebral spinal fluid), with any of the signs of clinical disease (e.g., sepsis, pneumonia, or meningitis). The presence of GBS in samples was determined within routine internal hospital laboratory services using the BD BACTEC^TM^ 9210 Culture System and API 20STREP (Guangzhou) and BD BACTEC^TM^ 9120 Blood Culture System and CAMP test (Changsha). GBS isolates were collected and stored in STGG stock medium at –70°C.

**Table 1:** *Streptococcus agalactiae* isolates used in this study.

Isolate name	Isolation source	Onset of disease	Isolation country	Reference	NCBI BioProject ID/Accession number
CH-1	CSF	LOD	Guangzhou	Liu et al., 2015	PRJNA324749
CH-2	blood	LOD	Guangzhou	Liu et al., 2015	PRJNA324749
CH-3	blood	EOD	Changsha	Liu et al., 2015	
CH-4	CSF	LOD	Guangzhou	Liu et al., 2015	
CH-5	blood	LOD	Guangzhou	Liu et al., 2015	
CH-6	blood	EOD	Guangzhou	Liu et al., 2015	
CH-7	CSF	LOD	Guangzhou	Liu et al., 2015	
CH-8	blood	LOD	Guangzhou	Liu et al., 2015	
GZ01	Blood	EOD	Guangzhou	This study	PRJNA324749
GZ02	Blood	EOD	Guangzhou	This study	PRJNA324749
GZ04	CSF	LOD	Guangzhou	This study	
GZ05	Blood	EOD	Guangzhou	This study	PRJNA324749
GZ06	Blood	LOD	Guangzhou	This study	PRJNA324749
GZ07	Blood	EOD	Guangzhou	This study	
GZ08	Blood	EOD	Guangzhou	This study	PRJNA324749
GZ09	Blood	EOD	Guangzhou	This study	
GZ11	Blood	LOD	Guangzhou	This study	PRJNA324749
GZ12	Blood	LOD	Guangzhou	This study	PRJNA324749
GZ13	Blood	LOD	Guangzhou	This study	PRJNA324749
GZ14	CSF	LOD	Guangzhou	This study	PRJNA324749
GZ15	Uri	LOD	Guangzhou	This study	
GZ16	Blood	LOD	Guangzhou	This study	
GZ18	Blood	LOD	Guangzhou	This study	
GZ19	Blood	LOD	Guangzhou	This study	PRJNA324749
GZ20	CSF	LOD	Guangzhou	This study	PRJNA324749
CS01	Blood	LOD	Changsha	This study	PRJNA324749
COH1	Blood		USA	Wilson and Weaver, 1985	HG939456

*CSF, cerebrospinal fluid; EOD, early-onset disease (0 to <7 days); LOD, late-onset disease (7–90 days); Uri, urine.*

*S. agalactiae* strains were grown at 37°C in 5% CO_2_ in Todd Hewitt Broth (Difco Laboratories), in trypticase soy-agar or Muller-Hinton-agar supplemented with 5% sheep blood.

The study protocols were approved by the Novartis Vaccines Research Institutional Board and by the Medical Ethical Committees of Guangzhou Women and Children’s Medical Center and of Changsha Hospital for Maternal and Child Health. The study was conducted in accordance with guidelines for Good Pharmacoepidemiology Practices and the Declaration of Helsinki. Informed consent was obtained from all subjects.

### Strain Serotyping by Latex Agglutination

Serotyping was performed using the standard Strep-B-Latex rapid agglutination method (Strep-B-Latex kit; Statens Serum Institut, Denmark). A heavy suspension of the test organism was prepared by harvesting bacteria from the agar plate in 250 μl of phosphate-buffered saline (PBS, pH 7.4). A 20 μl aliquot of the bacterial suspension was mixed with 5 μl of each of the ten Latex reagents (rabbit antisera Ia, Ib, and II–IX) onto a disposable card. The reaction card was rotated slowly and a positive reaction was indicated by agglutination appearing within 30 s.

### Antimicrobial Susceptibility Test

The *E* test strips (Biomiereux) method was used to assess the antimicrobial susceptibility by determining the minimal inhibitory concentrations (MICs) of these antibiotics: azithromycin, benzylpenicillin, clindamycin, erythromycin, levofloxacin, ceftriaxone, claritromycin, tetracycline, vancomycin. The breakpoints adopted were in accordance with the 2015 criteria set by the European committee on antimicrobial susceptibility testing based on tables for interpretation of MICs version 5.0^1^. The susceptibility for kanamycin, gentamycin, streptomycin, lincomycin, and spectinomycin was defined by Société Française de Microbiologie^2^.

### Genomic DNA Preparation

Genomic DNA was prepared from overnight GBS cultures by a standard protocol for Gram-Positive bacteria, using Mutanolysin-treatment of bacterial cells and a Gene Elute Bacterial Genomic DNA kit (Sigma–Aldrich) according to the manufacturer’s instructions.

### Molecular Analysis by PCR-Based Assays

A molecular capsular genotyping was used to confirm the Latex serotype assignment and to identify the capsular type of GBS isolates resulting serologically non-typeable (NT). The assay was performed by multiplex PCRs using a previously published method (Poyart et al., 2007) modified in house by including a new primer pairs specific for serotype IX detection (5′-AGATCATTTGTTCCTGATTCCCTAAAG-3′; 5′-AATCATCTTCATAATTTATCTCCCATT-3′). The pattern for type IX strains detected two amplicons (371 and 1,132 base pairs). The protocol is based on two multiplex PCRs, one containing the primer pairs specific for serotypes Ia, Ib, II, III, and IV and the other containing the primer pairs specific for serotypes V, VI, VII, VIII, and IX.

Pilus genes were PCR-amplified using primers specifically annealed to conserved genomic regions external to the coding sequences as already described (Margarit et al., 2009).

The presence of the Gbs2018/*hvgA* gene was assessed as previously described (Lamy et al., 2006; Tazi et al., 2010).

### Whole Genome Sequencing (WGS), Genome Assembly, and Annotation

Whole-genome sequencing was performed on libraries generated using 1 ng of purified bacterial DNA processed by Nextera XT DNA Library Preparation Kit (Illumina). Samples were normalized with a bead-based approach, pooled in an equal amount and finally diluted for the deep sequencing run, following Nextera XT protocol. Sequencing was carried out on a HiSeq 2500 platform in a 100 bp paired-end run with the TruSeq SBS version 3 chemistry (Illumina).

The newly sequenced genomes were assembled using SPAdes 3.0.0 (Bankevich et al., 2012). The contigs number ranged between 38 and 54, with an average of 45. All obtained draft genome assemblies were annotated with Prokka (Seemann, 2014) using a genus specific non-redundant BLAST database, built from all the translated annotations retrieved from RefSeq (Pruitt et al., 2009) for TaxId 1311, clustered with CD-HIT (Fu et al., 2012). The newly sequenced genomes were deposited in the NCBI database under the BioProject ID PRJNA324749.

### Genome Alignments and Sequence Analysis

The complete genome of GBS COH1 (Accession number HG939456) was used as reference sequence for genome analysis. Genome comparison and visualization was performed by BRIG software (Alikhan et al., 2011).

Sequence types were assigned by comparing the genomic sequences with the seven housekeeping genes of the GBS MLST (Multi Locus Sequence Type) system (Jones et al., 2003)^3^ using the Bio-MLST-Check-2.0.1510612 suite^4^. Clonal Complexes (CCs) were assigned as previously reported (Rosini et al., 2015). ICEs sequence alignments and comparison were performed using Easyfig (Sullivan et al., 2011). ICE sequence similarities were searched on the ICEberg server (Bi et al., 2012). ICESag(RR1) from CH-2 isolate was used as reference sequence for reads mapping and subsequent sequence comparison of the ICEs from all GBS genomes. Reads mapping and multiple alignments were performed using Geneious R9 with default settings^5^. The distribution of the antibiotic resistance genes present in the Antibiotic Resistance Database (Liu and Pop, 2009) and the RESFAMS database (Gibson et al., 2015) within the genomes of the 14 newly sequenced isolates was investigated using custom scripts based on the BLAST and HMMer algorithms.

## Results

### Most Analyzed GBS Isolates Are Associated to ST-17 and Carry Only Pilus Island 2b

To investigate the genetic characteristics of a clinically relevant GBS population from Southern China, we collected 26 isolates from infants affected by sepsis and meningitis in Guangzhou/Changsha area. Eight strains were obtained from EOD cases and 18 from neonates with LOD.

We first assessed the CPS type and the pilus islands (PIs) distribution by serological and PCR-based assays. Serotype III was the most prevalent accounting for 58% of all isolates (15/26) among both EOD (4/8) and LOD (11/18), followed by type Ib (19%; 5/26), type V (11%; 3/26), and type Ia (8%; 2/26). One genotype II strain was NT by serological analysis (4%; 1/26) (**Table 2**).

**Table 2:** Characteristics of the 26 GBS neonatal invasive isolates recovered in China.

	Capsular polysaccharide (CPS) type					
	Ia	Ib	II	III	V	Total
**Disease**						
EOD	1	2		4	1	8
LOD	1	3	1	11	2	18
**Pilus Islands**						
PI-2a	2	2				4
PI-2b				13		13
PI-1+2a		3	1	1	3	8
PI-1+2b				1		1
***hvgA***						
*hvgA–*	2	5	1	1	3	12
*hvgA+*				14		14

*Data are number of isolates. EOD, early onset disease; LOD, late onset disease; PI, pilus island; *hvgA (Gbs2018C)*, hypervirulent GBS adhesion gene.*

To verify whether the prevalent serotype III was associated to the hypervirulent ST-17 lineage as in previous observations related to the same region (Wang et al., 2015a), the isolates were screened for the presence of the *hvgA* gene, a characteristic feature of the ST-17 strains (Lamy et al., 2006). PCR results showed that 14 strains, all CPS type III, were *hvgA* positive and thus ST-17-associated. Only one type III isolate was negative for the presence of the gene and, as expected, the same was true for all strains belonging to other serotypes.

Concerning PIs distribution, half of the analyzed isolates only harbored PI-2b in their genomes (13 out of 26 strains). Moreover, all these isolates presenting the PI-2b-alone genotype belonged to CPS type III-ST-17. The PI-1+2a combination was present in eight strains distributed among four CPS types (three for type Ib and V each, one for type II and III). Finally, PI-2a alone was found in the four strains belonging to CPS types Ia or Ib (**Table 2**).

In conclusion, the vast majority of type III strains (14/15) belonged to the ST-17 lineage, and all but one carried the PI-2b alone, an uncommon genotype among type III-ST-17 isolates characterized so far (Springman et al., 2014).

### Whole Genome Sequencing Analysis Confirmed that Type III Chinese Isolates Belong to CC17

**FIGURE 1:**
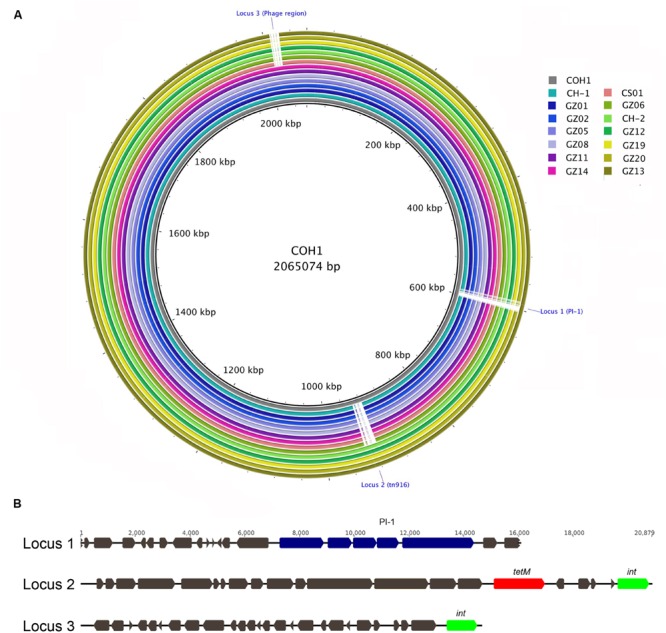
**Multi whole-genome comparison. (A)** The complete group B *Streptococcus* (GBS) COH1 (HG939456) genome was used as reference and compared against the 14 sequenced and assembled genomes. The innermost ring shows the COH1 genome length (kbp). The remaining rings show BLAST comparison of the 14 sequenced genomes: CH-1, GZ02, GZ05, GZ08, GZ11, GZ14, CS01, GZ06, CH-2, GZ12, GZ19, GZ20, and GZ113 from ring 2 to 15. Full colors represent a sequence identity over 99%. Interruptions in a colored ring indicate areas of the COH1 reference chromosome that are missing in the represented genomes (Locus 1, 2, and 3). **(B)** Schematic representation of GBS COH1 Locus 1 comprising pilus island genes (blue), Locus 2 including the *tn916* element carrying *tetM* gene (red), and the integrase gene *int* (green). A phage-related region is present in Locus 3.

To further investigate the molecular characteristics of the 14 identified *hvgA*-positive serotype III strains we performed WGS. *In silico* MLST analysis and CC assignment of the newly sequenced genomes confirmed that all strains belonged to CC17, and specifically 12 were strictly ST-17 while two had novel ST profiles that were single locus variants (s.l.v.) of ST-17. Further sequence analysis confirmed both the presence of the type III *cps* operon and the PI-2b in all strains and the absence of the otherwise well-conserved PI-1 in 13 of these 14 CC17 strains. To assess DNA sequence similarity, we performed BLAST pairwise sequence comparisons between each genome and the complete chromosome of the ST-17 reference strain COH1. **Figure 1** provides a comprehensive view of all single comparisons by displaying similarity between the central COH1 reference sequence and the other draft genomes as concentric rings. All analyzed genomes shared more than 99% sequence identity to the reference genome across at least 94% of its whole length. We also identified three distinct genomic loci in the COH1 reference chromosome (locus 1, 2, and 3) that could not be found in some of the newly sequenced strains. These regions are indicated by interruptions in the circular map that represent each genome (**Figure 1A**). Locus 1 is absent in all analyzed genomes except for one, while locus 2 and locus 3 are absent in 9 and 7 genomes, respectively. According to the curated annotation of the COH1 genome, locus 1 includes the entire pilus island 1 plus 15 genes belonging to different functional categories (Supplementary Table 1). Locus 2 and 3 mainly contain predicted open reading frames belonging to mobile elements along with poorly characterized hypothetical genes. Particularly, locus 2 includes a well-known large integrative conjugative element (ICE) Tn916 carrying the *tetM* gene conferring resistance to tetracycline (Da Cunha et al., 2014), while locus 3 hosts a putative prophage carrying different genes of unknown function (**Figure 1B**).

### CPS Type III CC17 Chinese isolates Have Acquired Multi-Drug Resistance Gene Clusters

**FIGURE 2:**
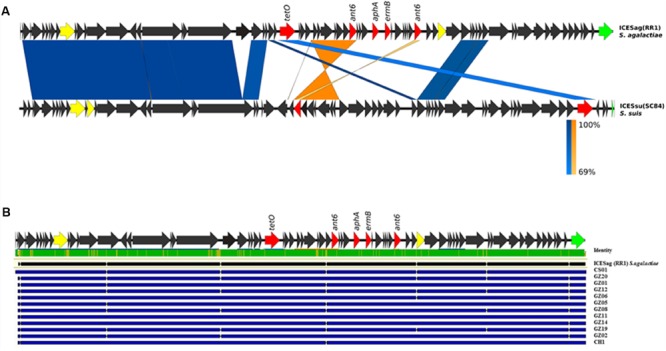
**Schematic representation of the newly identified integrative and conjugative elements (ICEs) in GBS genomes. (A)** Structural comparison of ICESag (RR1) with the corresponding regions present in ICESsu (SC84) from *Streptococcus suis*. Coding sequences (CDSs) of the newly identified ICESag(RR1) in GBS CH-2 isolate are depicted by arrows. Antibiotic resistance determinants are colored in red, conjugative type IV elements in yellow and integrase function in green. Blue bands indicate BLAST matches between sequences in the same orientation and orange twisted bands indicate BLAST matches between sequences in opposite orientations. In both cases, the intensity of the colors indicates the strength of the match. **(B)** Sequence comparison of the newly identified ICEs by multiple alignment. Blue lines depict single ICE sequences. Upper green bar represents the resulting sequence identity (green 100% identity, yellow <100% identity).

Horizontal gene transfer events determining exchange of large genome tracts are commonly considered an important mechanism in the diversification of *S. agalactiae* (Brochet et al., 2008). Therefore, we hypothesized that the loss in 13 out of 14 sequenced isolates of the genomic region corresponding to locus 1 could be counterbalanced by the acquisition of new genetic material, giving a potential selective advantage to these pathogenic strains. To test this hypothesis, the sequenced draft genomes were analyzed to look for new genes absent in the reference strain. The assembly of the sequenced short reads permitted to partially rebuild the regions corresponding to locus 1 for all 13 strains. We identified a large putative integrative and conjugative element (ICE), ICESag(RR1) in the PI-1 minus genomes replacing the entire locus 1. For one genome, namely CH-2, we were able to isolate the full-length ICE since it was harbored in a single sequence contig. This mobile element presented a typical mosaic organization, composed by different modules that showed a high sequence similarity to other ICEs found in different other streptococcal species such as *S. suis*, *S. dysgalactiae, S. pyogenes*, *S. pneumoniae.* Among all the ICEs present in the ICEberg database (Bi et al., 2012), the newly identified ICESag(RR1) displayed the highest sequence similarity (91% identity in a 5772 bp region) to *S. suis* ICESsu(SC84). More detailed sequence analysis revealed that the central module of this large mobile element contained several ORFs annotated as putative antibiotic resistance genes, including *tetO*, *ermB*, *ant6*, and *aphA*, known to confer multiple resistances to tetracycline, erythromycin and aminoglycosides (**Figure 2A**). Mapping of the reads of the remaining 12 strains lacking locus 1 on the extracted ICE revealed that the sequence identity of this tract was highly conserved in each strain (>98%) (**Figure 2B**). Finally, in strains lacking locus 2 or locus 3 we observed no additional genetic elements in the corresponding regions.

### Identification of a New Genetic Module Carrying Additional Antibiotic Resistance Genes

**FIGURE 3:**
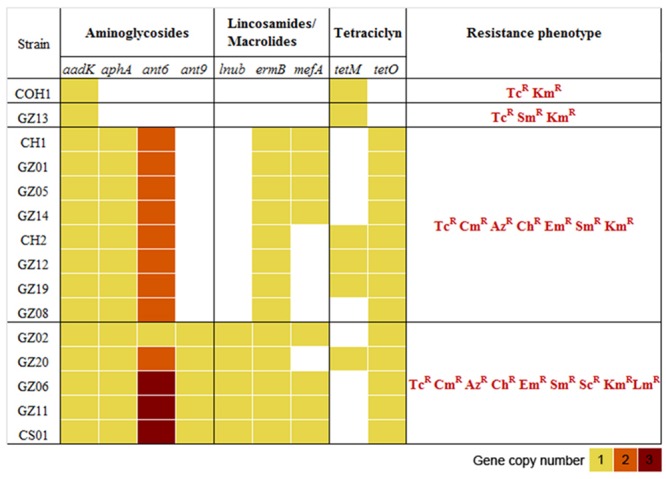
**Distribution of antibiotic resistance genes and phenotypes in the 14 GBS isolates selected for whole genome sequencing (WGS).** Heatmap representing the copy number of antibiotic resistance genes present in each genome. The antimicrobial susceptibility was assessed by *E* test determining the minimal inhibitory concentrations (MICs) to streptomycin (Sm), spectinomycin (Sc), kanamycin (Km), azithromycin (Az), claritromycin (Ch), erythromycin (Em), clindamycin (Cm), tetracycline (Tc), lincomycin (Lm), levofloxacin (Le), gentamycin (Gm), benzylpenicillin (Pg), ceftriaxone (Tx), vancomycin (Va). R, antibiotic resistant. All strains were susceptible to penicillin, ceftriaxone, vancomycin, levofloxacin, and gentamycin. COH1 was used as reference genome in the *in silico* resistome analysis and as reference strain in the antimicrobial susceptibility tests.

**FIGURE 4:**
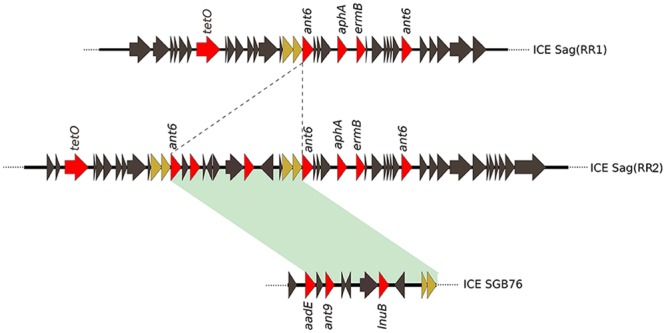
**Sequence comparison of the newly identified ICESag (RR2) and ICE SGB76 from *S. agalactiae*.** An example of composite ICESag (RR2) found in GBS GZ02, GZ20, GZ06, GZ11, and CS01 isolates. Coding sequences (CDSs) are depicted by arrows. Dotted lines represent the region of insertion of a new genetic module resembling the GBS ICE SGB76 carrying antibiotic resistance genes (red arrows). Green shadowing indicates 99% of sequence identity. Brown arrows indicate genes duplicated in ICESag (RR2).

The previous analysis showed that the invasive strains of our collection could be resistant to multiple antimicrobial compounds due to the acquisition of an uncommonly large number of resistance genes hosted on mobile elements. To gain a clearer picture of the antibiotic resistance gene repertoire of these strains we performed a deep search of their genomes using a custom approach integrating different antimicrobial resistance gene databases. As shown in **Figure 3**, all sequenced genomes harbored genes predicted to confer resistance against aminoglycosides and tetracycline antibiotics. Moreover, with the exception of GZ13, all sequenced isolates hosted at least one macrolide resistance gene. In most cases a single copy of each antibiotic resistance gene was detected, while two or three copies of *ant6* were found in all strains, except for GZ02 that carried a single copy. Moreover two additional genes predicted to confer resistance to spectinomycin and lincomycin, *ant9* and *lnuB*, were identified in a subgroup of strains (GZ02, GZ20, GZ06, GZ11, and CS01). These genes were contained in a new gene module similar to the previously described ICE SGB76 (Montilla et al., 2014), that was found inserted in the ICE Sag(RR1), thus resulting in a previously undescribed ICE configuration that was named Sag(RR2) (**Figure 4**).

### Antimicrobial Susceptibility Analysis Confirmed *In silico*-Predicted Multi-Drug Resistance

To verify whether the identified genes were able to confer antibiotic resistance to the strains harboring them, we tested susceptibility on plates to 14 different chemical compounds belonging to different antibiotic classes (benzylpenicillin, ceftriaxone, streptomycin, spectinomycin, kanamycin, azithromycin, claritromycin, erythromycin, clindamycin, lincomycin, tetracycline, levofloxacin, gentamycin, and vancomycin). The results confirmed that the antibiotic resistance profiles predicted *in silico* closely matched the resistance phenotype observed *in vivo* (**Figure 3**). Overall, these results revealed a direct correlation between the presence of a specific antibiotic resistance gene and a resistance phenotype. Compared to COH1 phenotype, 13 CC17 Chinese strains showed additional antibiotic resistance to macrolides and lincosamides. Moreover, the subgroup including the strains carrying the *ant9* and *lnuB* genes showed also resistance to lincomycin and spectinomycin. Conversely, benzylpenicillin, ceftriaxone, levofloxacin, gentamycin, and vancomycin proved to be effective in preventing growth of all isolates.

## Discussion

This study reports the first whole-genome sequencing analysis of GBS clinical isolates collected in China from neonates with invasive disease. To date, only few surveillance studies on GBS infections have been carried out in China, and in general, in Asia, so the burden of disease caused by GBS in infants younger than 90 days is still unclear. Moreover, data related to serotype distribution and genetic characteristics of the prevalent circulating lineages in this particular geographical area are currently rare, although critical for a deep understanding of the global epidemiology of GBS infections.

Among the 26 neonatal isolates analyzed in this study, we found that most were serotype III, and all type III except one belonged to the well-known hypervirulent CC17 lineage (Musser et al., 1989). Wang et al. (2015a) recently reported the prevalence of this specific lineage (CC17-serotype III) among GBS isolates causing neonatal disease in China, and it is noteworthy that those isolates also showed resistance to erythromycin, clindamycin, and tetracycline. Serotype III strains represent the most common cause of GBS neonatal infections worldwide (Edmond et al., 2012; Le Doare and Heath, 2013), and the spread of type III strains, exhibiting increased antibiotic resistance, appears clearly higher in China than in other countries. Therefore, to investigate the high prevalence of CC17 strains in this geographic area, we analyzed by WGS 14 type III-CC17 clinical isolates from infected neonates collected in the Chinese area. Recent comparative genomics and phylogenetic analysis have confirmed that isolates belonging to CC17 have closely similar evolutionary histories and their observed homogeneity is due to the relatively low rate of recombination among strains respect to the other CCs (Da Cunha et al., 2014; Teatero et al., 2016). Consistently, our genome analysis has confirmed a high degree of genetic identity in the GBS CC17 population. Additionally, it has led us to the identification of multi-drug resistance gene clusters harbored in at least two different versions of ICEs, carrying five or eight antibiotic resistance genes, respectively. The acquisition of these ICEs occurred in a specific genomic locus in 13 out of 14 CC17 isolates analyzed, and balanced the loss of a 16 kb genome region that include the entire pathogenicity pilus island 1 in the ST-17 reference genome.

Our findings are in agreement and expand recently published observations by Teatero et al. (2016), who identified 11 CC17 Canadian isolates lacking PI-1 and acquiring in the same location a mobile genetic element (MGE) encoding resistance to tetracycline, macrolides and other antibiotics. Until these discoveries, human-derived CC17 isolates were exclusively associated with the simultaneous presence of pilus islands 1 and 2b (Springman et al., 2014). Interestingly, a recent study investigating PI distribution on 160 GBS isolates collected from colonized pregnant women in six hospital settings in Beijing, 10 out of 14 CC17 isolates contained PI-2b alone (Lu et al., 2015). Although not confirmed by genome analysis, it is presumable that also these carrier strains have acquired new genic elements replacing the PI-1 locus.

These observations lead us to speculate that trading an important pathogenicity locus for a multi-drug resistance mobile element could represent one of the causes for the high expansion of the CC17 lineage among Chinese isolates.

It is known that ICEs are highly variable and could easily lose or acquire modules (Wozniak and Waldor, 2010). In our small collection of neonatal isolates, we found a surprisingly high number of different genes conferring resistance to different antibiotic classes. These mobile elements are inserted in a specific genome locus and could acquire additional resistance genes by recombining their modules. Indeed, it is really interesting to note that the module containing the *ant9* and *lnuB* genes for the resistance to lincomycin and spectinomycin was originally identified in a GBS pathogenic strain isolated in Argentina (Montilla et al., 2014), a remote location from the region of isolation of the strains analyzed in this study.

Antimicrobial resistance has been shown to be emerging worldwide in the GBS population, and in particular, resistance to clindamycin and erythromycin is increasing (Castor et al., 2008; CDC, 2012). While none of the 15 analyzed isolates were resistant to penicillin, vancomycin or ceftriaxone, all of them were resistant to tetracycline. In addition, all strains harboring the identified ICEs were resistant to macrolides (claritromycin, azithromycin, and erythromycin) and kanamycin. Among these, the subgroup carrying the *ant9* and *lnuB* genes showed also resistance to spectinomycin and lincomycin. To date, treatment of GBS infections is mainly based on intra-partum prophylaxis with penicillin, ampicillin, and cefazolin; second-line drugs such as vancomycin, macrolides (i.e., erythromycin, azithromycin, and clarithromycin), and lincosamides (clindamycin and lincomycin) may be used as alternative in patients allergic to penicillin or cephalosporins. To date, rare cases of invasive vancomycin-resistant GBS strains have been isolated from adult patients (Park et al., 2014). However, the Chinese scenario is of great concern, since the common usage of human antibiotics in the veterinary sector in the past decades has led to high incidence of multidrug resistant bacteria (Cheng et al., 2015). The frequency of resistance to clindamycin and erythromycin among circulating GBS isolates in China has been reported to be significantly higher than in other countries (Wang et al., 2015a,b).

All together these observations underline the importance of genomic surveillance of epidemiologically relevant lineages and show the power of the *in silico* analysis of the GBS resistome to predict antibiotic resistance phenotypes and to understand the epidemiology and plasticity of GBS population. Moreover, even though effective drugs are still available to tackle the majority of GBS infections, the high plasticity of the MGE carrying multiple antibiotic resistance genes and the advantage they confer to pathogens in countries with an extensive antibiotic use, such as China, underline the need to develop preventive approaches based on vaccination.

## Author Contributions

EC, RR, CN, IM, and CR conceived and designed the study. EC, RR, SG, and CR performed the experiments. EC, RR, MR-L, and CR analyzed data. WJ, GG, QD, HZ, WW, and HL identified and collected GBS isolates. EC, RR, IM, and CR wrote the manuscript. All authors reviewed and approved the manuscript.

## Conflict of Interest Statement

RR, SG, WJ, GG, CN, IM, and CR, were all permanent employees of Novartis (now GSK Vaccines) at the time of the study. The authors declare that Novartis/GSK provided support in the form of salaries. All the other authors declare that the research was conducted in the absence of any commercial or financial relationships that could be construed as a potential conflict of interest.

## Footnotes

1 http://www.eucast.org

2 http://www.sfm-microbiologie.org

3 http://pubmlst.org/sagalactiae/

4 http://search.cpan.org/~{}ajpage/Bio-MLST-Check-2.0.1510612/

5 http://www.geneious.com
