# Supplementary material for: Genomic Analysis Reveals Multi-Drug Resistance Clusters in Group B Streptococcus CC17 Hypervirulent Isolates Causing Neonatal Invasive Disease in Southern Mainland China
Source: Front Microbiol. 2016 Aug 15;7:1265. doi: 10.3389/fmicb.2016.01265 (PMC4983569; doi:10.3389/fmicb.2016.01265)
Supplement: Supplementary file 1 [file Table_1.PDF]

Supplementary Material

Genomic analysis reveals multi-drug resistance clusters in Group B Streptococcus CC17 hypervirulent isolates causing neonatal invasive disease in China

Edmondo Campisi, Roberto Rosini*, Wenjing Ji, Silvia Guidotti, Maricarmen Rojas-López, Guozhu Geng, Qiulian Deng, Huamin Zhong, Weidong Wang, Haiying Liu, Cassandra Nan, Immaculada Margarit, C. Daniela Rinaudo*

*** Correspondence:**
C. Daniela Rinaudo, daniela.x.rinaudo@gsk.com
Roberto Rosini, roberto.x.rosini@gsk.com

Supplementary Table 1

Annotation in COH1 genome (Accession number HG939456) of the genes present in locus 1, locus 2 and locus 3 represented in Figure 1.

**Locus 1**								
**Locus_tag**	**Protein Function\Description**	**Minimum**	**Maximum**	**Length (bp)**	**Min (original sequence)**	**Max (original sequence)**	**Direction**	**Description (Figure 1)**
**GBSCOH1_0554**	RNA methyltransferase, TrmA family	1	31	31	587.464	587.494	forward	
**GBSCOH1_0555**	Unknown-related protein	139	360	222	587.602	587.823	forward	
**GBSCOH1_0556**	HAD superfamily (subfamily IIIB) phosphatase,TIGR01672	478	1.215	738	587.941	588.678	forward	
**GBSCOH1_0557**	hypothetical protein	1.535	2.053	519	588.998	589.516	forward	
**GBSCOH1_0558**	conserved hypothetical protein	2.181	2.417	237	589.644	589.880	reverse	
**GBSCOH1_0559**	Transcriptional regulator, TetR family	2.398	2.706	309	589.861	590.169	reverse	
**GBSCOH1_0560**	cell wall surface anchor family protein,truncation	2.889	3.221	333	590.352	590.684	forward	
**GBSCOH1_0561**	transposase OrfB, IS3 family, truncation	3.342	4.091	750	590.805	591.554	reverse	
**GBSCOH1_0562**	probable transposase TnpA	4.211	4.486	276	591.674	591.949	reverse	
**GBSCOH1_0563**	Tn5252, Orf 10 protein	4.607	4.756	150	592.070	592.219	forward	
**GBSCOH1_0564**	hypothetical protein	4.820	4.939	120	592.283	592.402	forward	
**GBSCOH1_0565**	chaperonin, 33 kDa	4.977	5.180	204	592.440	592.643	reverse	
**GBSCOH1_0566**	chaperonin, 33 kDa	5.144	5.491	348	592.607	592.954	reverse	
**GBSCOH1_0567**	transcriptional regulator, AraC family	5.685	6.893	1.209	593.148	594.356	reverse	
**GBSCOH1_0568**	cell wall surface anchor family protein	7.274	8.938	1.665	594.737	596.401	forward	PI-1
**GBSCOH1_0569**	cell wall surface anchor family protein	9.027	9.950	924	596.490	597.413	forward	PI-1
**GBSCOH1_0570**	sortase family protein	9.952	10.869	918	597.415	598.332	forward	PI-1
**GBSCOH1_0571**	sortase family protein	10.826	11.677	852	598.289	599.140	forward	PI-1
**GBSCOH1_0572**	cell wall surface anchor family protein,putative	11.759	14.431	2.673	599.222	601.894	forward	PI-1
**GBSCOH1_0573**	sortase family protein	14.706	15.275	570	602.169	602.738	forward	
**GBSCOH1_0574**	protein of unknown function	15.491	16093	603	602.954	603.556	forward	

**Locus 2**								
**Locus_tag**	**Product**	**Minimum**	**Maximum**	**Length**	**Min (original sequence)**	**Max (original sequence)**	**Direction**	**Description (Figure 1)**
**GBSCOH1_0864**	uncharacteried conserved protein CAC1439	577	891	315	916.026	916.340	forward	
**GBSCOH1_0865**	conserved hypothetical protein	912	1.289	378	916.361	916.738	forward	
**GBSCOH1_0866**	conserved hypothetical protein	1.299	2.072	774	916.748	917.521	forward	
**GBSCOH1_0867**	TN916 ORF21 homolog lmo1112	2.094	3.497	1.404	917.543	918.946	forward	
**GBSCOH1_0868**	transcriptional regulator, Cro /CI family	3.679	4.863	1.185	919.128	920.312	forward	
**GBSCOH1_0869**	conserved hypothetical protein	4.860	5.132	273	920.309	920.581	forward	
**GBSCOH1_0870**	conserved hypothetical protein	5.129	5.350	222	920.578	920.799	forward	
**GBSCOH1_0871**	conserved hypothetical protein	5.410	6.171	762	920.859	921.620	forward	
**GBSCOH1_0872**	TN916 ORF18 homolog lmo1108	6.233	6.733	501	921.682	922.182	forward	
**GBSCOH1_0873**	hypothetical protein	6.805	7.830	1.026	922.254	923.279	forward	
**GBSCOH1_0874**	TN916 ORF17 homolog lmo1107	7.859	8.296	438	923.308	923.745	forward	
**GBSCOH1_0875**	ATP /GTP-binding protein, putative	8.280	10.733	2.454	923.729	926.182	forward	
**GBSCOH1_0876**	membrane protein, putative	10.730	12.757	2.028	926.179	928.206	forward	
**GBSCOH1_0877**	TN916 ORF14 and to L. monocytogenes P60 protein homolog lmo1104	12.754	13.776	1.023	928.203	929.225	forward	
**GBSCOH1_0878**	TN916 ORF13 homolog lmo1103	13.793	14.722	930	929.242	930.171	forward	
**GBSCOH1_0879**	tetracycline resistance protein	15.099	17.018	1.920	930.548	932.467	forward	TetM
**GBSCOH1_0880**	Tn916, transcriptional regulator, putative	17.362	17.715	354	932.811	933.164	reverse	
**GBSCOH1_0881**	putative sigma-70 family protein	18.187	18.669	483	933.636	934.118	forward	
**GBSCOH1_0882**	conserved hypothetical protein	18.666	18.896	231	934.115	934.345	forward	
**GBSCOH1_0883**	conserved hypothetical protein	19.392	19.592	201	934.841	935.041	forward	
**GBSCOH1_0884**	site-specific recombinase, phage integrase family	19.619	20.812	1.194	935.068	936.261	forward	Integrase

Locus 3								
**Locus_tag**	**Product**	**Minimum**	**Maximum**	**Length**	**Min (original sequence)**	**Max (original sequence)**	**Direction**	**Description (Figure 1)**
**GBSCOH1_1937**	hypothetical protein	463	1.086	624	2.009.405	2.010.028	reverse	
**GBSCOH1_1938**	conserved hypothetical protein	1.076	1.582	507	2.010.018	2.010.524	reverse	
**GBSCOH1_1939**	hypothetical protein	1.785	1.943	159	2.010.727	2.010.885	forward	
**GBSCOH1_1940**	Phage encoded transcriptional regulator, ArpU family	2.076	2.462	387	2.011.018	2.011.404	reverse	
**GBSCOH1_1941**	conserved hypothetical protein	2.437	2.799	363	2.011.379	2.011.741	reverse	
**GBSCOH1_1942**	hypothetical protein	2.823	3.002	180	2.011.765	2.011.944	reverse	
**GBSCOH1_1943**	conserved hypothetical protein	3.198	3.686	489	2.012.140	2.012.628	reverse	
**GBSCOH1_1944**	conserved hypothetical protein	3.771	4.376	606	2.012.713	2.013.318	reverse	
**GBSCOH1_1945**	DnaD and phage-associated domain protein	4.457	5.278	822	2.013.399	2.014.220	reverse	
**GBSCOH1_1946**	hypothetical protein	5.447	5.719	273	2.014.389	2.014.661	reverse	
**GBSCOH1_1947**	conserved hypothetical protein	5.712	6.053	342	2.014.654	2.014.995	reverse	
**GBSCOH1_1948**	conserved hypothetical protein	6.050	6.412	363	2.014.992	2.015.354	reverse	
**GBSCOH1_1949**	conserved hypothetical protein	6.424	6.618	195	2.015.366	2.015.560	reverse	
**GBSCOH1_1950**	conserved hypothetical protein	6.615	6.947	333	2.015.557	2.015.889	reverse	
**GBSCOH1_1951**	hypothetical protein	7.036	7.542	507	2.015.978	2.016.484	reverse	
**GBSCOH1_1952**	conserved hypothetical protein, copG family transriptional regulator	7.631	7.804	174	2.016.573	2.016.746	reverse	
**GBSCOH1_1953**	BRO family, N-terminal domain protein	7.903	8.541	639	2.016.845	2.017.483	reverse	
**GBSCOH1_1954**	conserved hypothetical protein	8.764	9.006	243	2.017.706	2.017.948	reverse	
**GBSCOH1_1955**	BRO family, N-terminal domain protein	9.033	9.653	621	2.017.975	2.018.595	reverse	
**GBSCOH1_1956**	putative transcription regulator	9.677	9.877	201	2.018.619	2.018.819	reverse	
**GBSCOH1_1957**	hypothetical protein	10.083	10.697	615	2.019.025	2.019.639	forward	
**GBSCOH1_1958**	hypothetical protein	10.709	10.996	288	2.019.651	2.019.938	forward	
**GBSCOH1_1959**	hypothetical protein	11.453	11.695	243	2.020.395	2.020.637	forward	
**GBSCOH1_1960**	conserved hypothetical protein	11.707	12.048	342	2.020.649	2.020.990	forward	
**GBSCOH1_1961**	conserved hypothetical protein	12.075	13.040	966	2.021.017	2.021.982	forward	
**GBSCOH1_1962**	integrase /recombinase, phage integrase family	13.378	14.544	1.167	2.022.320	2.023.486	forward	Integrase
